# The effects of video-assisted breastfeeding education given to primiparous pregnant women on breastfeeding self-efficacy: randomized control study

**DOI:** 10.1186/s12884-024-06317-1

**Published:** 2024-02-17

**Authors:** Ayşe Metin, Nazlı Baltacı

**Affiliations:** 1 Faculty of Health Sciences, Department of Nursing, Erzurum Technical University, Erzurum, Turkey; 2 Faculty of Health Sciences, Department of Nursing, Ondokuz Mayıs University, Samsun, Turkey

**Keywords:** Pregnancy, Breastfeeding self-efficacy, Video-assisted education, Breastfeeding education

## Abstract

**Background:**

Breastfeeding is vitally important for the health of the mother, baby, family and society. Especially the perception of breastfeeding self-efficacy of primiparous pregnant women is an important factor in breastfeeding. This study was conducted to determine the effects of online video-supported breastfeeding education on breastfeeding self-efficacy in primiparous pregnant women.

**Methods:**

This randomized controlled study was conducted with primiparous pregnant women admitted to a university hospital in northern Turkey. The study involved 80 pregnant women, with 40 assigned to the experimental group and 40 to the control group. Participants in the intervention group received online video-assisted education, which covered the first meeting of the mother and baby as well as the initial breastfeeding session. The data for the breastfeeding self-efficacy scale were gathered at the onset of the study and three weeks later. In data analysis, categorical variables were assessed using the chi-square test, continuous variables and intergroup comparisons were conducted through the independent sample t-test, and intragroup comparisons were performed using the paired sample t-test.

**Results:**

While the baseline breastfeeding self-efficacy levels of the primiparous pregnant women were similar between the groups, statistically significant differences were observed both within (*p* = 0.000) and between (*p* = 0.000) groups in the breastfeeding self-efficacy scores of pregnant women in the intervention group after the education intervention.

**Conclusion:**

Breastfeeding self-efficacy levels in the education group showed a statistically significant increase compared to both the pre-education and control groups. This highlights the importance of nurses providing support to primiparous pregnant women through video-assisted education during pregnancy to enhance breastfeeding self-efficacy.

**Trial registration:**

ClinicalTrials.gov: NCT06121973 date of first registration (27/10/2023), retrospectively registered (08/11/2023).

## Introduction

Breastfeeding is vitally important for the health of the mother, the infant, the family and society as a whole [1]. It offers a myriad of benefits, including nutritional, immunological, psychological, social, economic, and environmental benefits. Furthermore, breastfeeding fosters a strong bond between mother and baby, reinforcing its significance in promoting overall well-being [2–4]. Breastfeeding insufficiency poses numerous challenges, with infant mortality being a prominent concern. Studies indicate that embracing universal breastfeeding could potentially avert 823,000 annual child deaths under 5 years of age and 20.000 maternal deaths due to breast cancer worldwide [4]. For this reason, numerous institutions and organizations, with the World Health Organization (WHO) at the forefront, emphasize the significance of breast milk and advocate exclusive breastfeeding for infants during the first 6 months [5]. Breastfeeding is prominently featured in the Sustainable Development Goals (SDGs), with a direct linked to “SDG 2 Ending Hunger, Food Security, and Better Nutrition Assurance” and “SDG 3 Ensure healthy lives and promote well-being for all at all ages” [6]. According to the report prepared by the United Nations Children’s Fund (UNICEF) and WHO as part of the Global Breastfeeding Partnership—a initiative working towards enhancing global breastfeeding rates— it is reported that no countries worldwide that fully adhere to breastfeeding standards [7]. Although breastfeeding is supported on many international platforms, globally, only half of new-born babies are breastfed within the first hour, and about one-third of babies in low- and middle-income countries are fed with water and animal milk before breastfeeding [7]. The burden of this situation on the economies of countries worldwide is estimated to be 341,3 billion USD annually [3]. The situation is no different in Turkey. National data for Turkey in 2018 shows that 41% of babies under 6 months of age receive only breast milk, and 71% are breastfed within the first hour after birth [8].

Many individual and socioeconomic factors affect the initiation and continuation of breastfeeding. Mothers often discontinue breastfeeding due to perceived difficulties rather than individual preference. Health professionals are to improve breastfeeding duration rates effectively, they need to identify and address predisposing factors that may contribute [9]. One possible modifiable variable is breastfeeding self-efficacy [9–11]. In Bandura’s cognitive-social theory, self-efficacy is a cognitive dynamic process that assesses people’s beliefs and their ability to conduct healthy behavior [12]. Breastfeeding self-efficacy is among the individual factors and is a crucial determinant of breastfeeding duration [13–16]. Perception of breastfeeding self-efficacy is the efficacy a mother feels she has about breastfeeding. It is also defined as a woman’s self-belief and confidence in her perceived breastfeeding ability [17]. Self-efficacious individuals tend to persist in a given task until they achieve success, whereas those who are self-inefficacious tend to give up prematurely [14].

Self-efficacy affects individuals’ expectations, behaviors, efforts, determination in the face of difficulties, and what cognitions they adopt that weaken or encourage them. The majority of women find the initial experience of breastfeeding to be painful, difficult, and challenging. Hence, some choose not to continue breastfeeding or discontinue it shortly after starting [18]. Considering the literature, pregnant women who are not confident in their ability to breastfeed are significantly more likely to stop breastfeeding before 2 weeks postpartum [19]. A systematic review showed that feeding the baby with only breast milk for the first 6 months postpartum was influenced by the mother’s breastfeeding self-efficacy [16]. On the other hand, it has been reported that women with higher breastfeeding self-efficacy levels are generally more successful in initiating and maintaining breastfeeding [16, 20]. Breastfeeding self-efficacy needs to be developed in all pregnant and postpartum women. However, primiparous pregnant women are more likely to encounter breastfeeding problems because they do not have any previous experience with breastfeeding. It has been reported that breastfeeding rates among primiparous pregnant women during the postpartum period are lower than those among multiparous pregnant women [21–23]. Women who have confidence in their ability to breastfeed successfully are better equipped to overcome barriers [14]. It is important to support breastfeeding self-confidence and breastfeeding determination in the face of difficulties by improving breastfeeding self-efficacy in primiparous pregnant women. Therefore, the mother needs to gain self-confidence by supporting breastfeeding self-efficacy in health care [24]. The woman’s confidence in breastfeeding is a very important issue that health professionals should take into account when supporting women in breastfeeding [3, 14]. Although breastfeeding education usually covers the postpartum period [25], pregnancy is an important period to prepare women for breastfeeding [26–28]. Women should receive breastfeeding education during pregnancy to successfully initiate and maintain breastfeeding in the postpartum period [23, 27, 29]. In a qualitative study conducted with prenatal and postpartum pregnant women, it is recommended that mothers need to be better educated for breastfeeding prenatally, and the information must be consistent, realistic, and evidence-based [30]. Training provided on breastfeeding during pregnancy increases women’s knowledge about breastfeeding and, at the same time, reinforces positive breastfeeding practices [31].

Today, the use of technology in breastfeeding education and motivation receives much more attention than traditional breastfeeding education. The use of web-based and video demonstration approaches in breastfeeding education programs is gradually increasing and being recommended more [28, 32]. Video-supported trainings are very important in that they appeal to many senses and support especially affective, cognitive and psychomotor learning. It creates an integrating and transformative learning process in learning. Therefore, the use of video in education needs to become widespread. This approach provides a value-added learning benefit by increasing the potential to improve attitudes towards breastfeeding support [28]. Verbal persuasion and transfer of factual information are likely used in breastfeeding support training. In video-supported trainings, the skill will be transferred directly and the woman will be able to build confidence that she can apply it [33]. Various studies in the literature report the development of breastfeeding self-efficacy during pregnancy [11, 20, 34]. When studies conducted were reviewed, no study was found in which online education was provided to primiparous pregnant women who had no previous experience in the prenatal or postpartum period by using a video demonstrating first mother-baby meeting and breastfeeding. It was predicted that self-efficacy would improve when the woman experiencing her first pregnancy mastered what she would experience in the process. In a systematic review published in 2024, Oggero et al. cited the lack of studies using virtual meeting environments or providing breastfeeding support via video-based telehealth as an important limitation of the current literature. In this context, the study will be beneficial as it is among the first studies with online video support in the field of breastfeeding [35]. For this reason, in this study, a video showing the first mother-baby meeting and breastfeeding on the first day of birth, which pregnant women can watch again whenever they want, was prepared and used by including it in the training content. The study aimed to determine the effects of online video-supported breastfeeding education on breastfeeding self-efficacy in primiparous pregnant women.

### Hypotheses

H0: Online video-assisted breastfeeding education does not change the breastfeeding self-efficacy levels of primiparous pregnant women.

H1: Online video-assisted breastfeeding education increases the breastfeeding self-efficacy levels of primiparous pregnant women.

## Methods

### Type of research

This research is a randomized controlled experimental study with a two-arm parallel pretest-posttest design. Pre- and post-tests were performed with the Prenatal Breastfeeding Self-Efficacy Scale [36]. The trial followed the CONSORT guidelines and noted at ClinicalTrials.gov ClinicalTrials.gov: NCT06121973 (27/10/2023), retrospectively registered.

### Participants

Population of the study consisted of pregnant women who were followed up in the obstetrics outpatient clinics of a university hospital in northern Turkey. A similar study in the literature [37] was utilized in order to calculate the sample size of the study, and as a result of the power analysis performed according to the margin of error of 0.05, beta value of 0.02 and 95% confidence interval, it was determined that at least 37 pregnant women should be included in each group. Each group included 40 pregnant women, considering the possible data loss in the study. Primiparous pregnant women who were in their second trimester, who did not have any risk in their pregnancy, who volunteered to participate in the study and who were literate were included in the study. Pregnant women who had auditory, visual, psychological or cognitive problems were excluded from the study. The research was conducted between June 1, 2022, and March 15, 2023, with pregnant women in the second trimester who were followed up in a university hospital.

### Randomization

Primiparous pregnant women were randomly allocated to the experimental group and the control group. Randomization was conducted using the https://www.graphpad.com/quickcalcs/randomize2/ program, which generated a list of random numbers. An independent statistician determined, before the study, the group assignment for pregnant women who met the inclusion criteria based on the order of their admission to the outpatient clinics.

### Blinding

In this study, the individuals who collected the data implemented the training program, and analyzed the data were different. The person who evaluated the data was also blinded to the experimental and control groups, thus a two-way blinding was provided.

### Procedures

The researcher evaluated the eligibility of the pregnant women admitted to the gynaecology outpatient clinics for pregnancy follow-up and the necessary information about the study was given to the pregnant women who were willing and eligible to participate. Written informed consents of these pregnant women were obtained and they were randomly divided into experimental and control groups. A pre-test was administered to both groups at the beginning of the study. Pregnant women in the experimental group received breastfeeding training synchronous in an online class (https://meet.google.com/zsd-ions-gsw) in groups of 6–8 people, formed according to the order of arrival. A total of 6 groups were created according to the order in which the pregnant women arrived at the hospital. Each of these groups attended training once a week for 3 weeks. During the same process, consultancy was provided individually via phone and WhatsApp applications. Four pregnant women benefited from individual counseling throughout the study. These pregnant women asked the educator questions about nipple problems and breastfeeding in various diseases. Group trainings were completed in 3 weeks with weekly sessions lasting 60–90 min. In the first session, online education was provided on breastfeeding physiology and the benefits of breastfeeding for mother, infant, family and society and the questions of pregnant women were answered. In the second session, questions of pregnant women were answered by providing online education on hunger and satiety symptoms of infants, milk stroke reflex, oxytocin massage, holding positions, milking and storage conditions. In the third session, online education was provided by using a video recording prepared by the researchers by using breast materials and a model baby. The meeting of mother and baby and the first breastfeeding were demonstrated in the video recording. This session was accompanied by a video demonstrating holding the baby, the baby’s grasping the breast, breastfeeding, burping the baby and breast care. The video was shared with the pregnant women in the intervention group so that they could use it continuously. Pregnant women in the control group received routine care and follow-up at the hospital and they received no intervention. Post-test was administered to the pregnant women three weeks after the pre-test. Data were collected face-to-face when the pregnant women came to the hospital for pregnancy follow-up without interfering with their routine care and follow-up. At the end of the study, the same education was given collectively to the pregnant women in the control group at an appropriate time.

### Instruments

Data were collected face-to-face by using “Personal Information Form” and “Prenatal Breastfeeding Self-Efficacy Scale”.

#### Personal information form

Personal information form, which included demographic and obstetric characteristics of the pregnant women, included a total of 19 questions developed by the researchers in line with the literature [1, 23, 26].

#### Prenatal breastfeeding self-efficacy scale [PBSES]

The scale was developed by Wells et al. to determine the breastfeeding self-efficacy perceptions of pregnant women in the prenatal period [38]. Turkish validity and reliability of the scale was conducted by Aydın and Pasinlioğlu [36]. The scale consists of a total of 20 items. Each item of the scale is a 5-point Likert scale ranging between “1 = Not at all sure- 5 = Completely sure”. Minimum 20 and maximum 100 points can be obtained from the scale. Higher score indicates breastfeeding self-efficacy perception. Cronbach’s alpha value of the scale was reported to be 0.86 [36]. Cronbach’s alpha value was found to be 0.96 in this study.

### Statistical analysis

Data were evaluated by using IBM SPSS v.23 [IBM Corp. Armonk, NY, USA) program and presented with descriptive statistics such as “number, percentage, arithmetic mean, standard deviation”. Chi-square test for categorical variables and independent sample t-test for continuous variables were used to confirm the differences in demographic and obstetric characteristics between groups. Independent sample t-test was used for intergroup comparison of PBSES scores and paired sample t-test was used for intragroup comparison. Statistical significance value was accepted as *p* < 0.05.

## Results

### Participant flow, demographic and obstetric characteristics

**Fig. 1:**
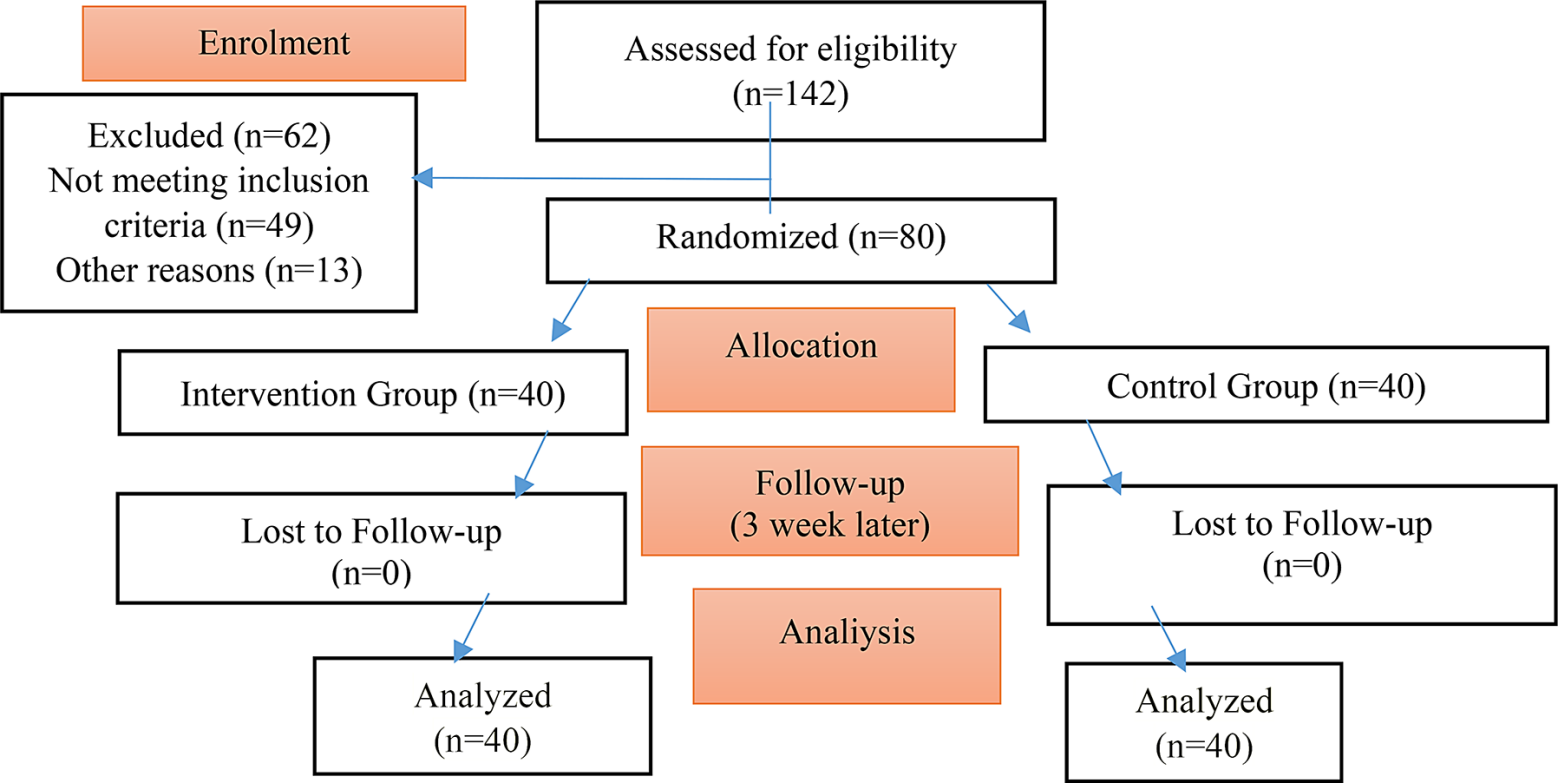
CONSORT flow chart

In the present study, 142 pregnant women who were admitted to obstetric outpatient clinics were evaluated for eligibility and 62 pregnant women were excluded. A total of 80 primiparous pregnant women, 40 in the experimental group and 40 in the control group, were randomized. No pregnant women withdrew from the study after randomization. In each group, 40 participants completed the study and they were included in the analysis. The CONSORT flow chart of the study is shown in Fig. 1.

No significant difference was found between the groups in terms of pregnant women’s age, duration of marriage, employment status, education and economic status, place of residence, gestational week, pregnancy plan, health problems during pregnancy, harmful habits and having social support, receiving breastfeeding education previously, thinking that they would breastfeed easily after birth, and their views about the importance of breastfeeding for their baby, themselves and the economy (*p* > 0.05) (Table 1).

According to the results, the education group (EG) and the control group (CG) were homogeneous in terms of many demographic and obstetric characteristics. In addition, pregnant women in the experimental group reported that they wanted to receive education, whereas those in the control group reported that they did not want to receive education. All pregnant women (*n* = 80). stated that they had regular pregnancy follow-ups and they had a nuclear family.

**Table 1:** Demographic and obstetrics characteristics between the groups

Characteristics	Education group (*n* = 40)	Control group (*n* = 40)	*p* value
Age (years)	28.20 (4.84)	26.42 (5.93)	0.147 ^a^
Length of marriage (years)	2.37 (1.51)	3.00 (3.86)	0.346 ^a^
Employment status			
Employed	18 (45.0)	11 (27.5)	0.163 ^b^
Unemployed	22 (55.0)	29 (72.5)	
Educational status			
Primary education	9 (22.5)	14 (35.0)	0.313 ^b^
Secondary education	18 (45.0)	12 (30.0)	
Higher education	13 (32.5)	14 (35.0)	
Economic situation			
Income equal to expense	32 (80.0)	35(87.5)	0.545 ^b^
Income higher than expense	8 (20.0)	5 (12.5)	
Place of residence			
Town	24 (60.0)	27 (67.5)	0.642 ^b^
City centre	16 (40.0)	13 (32.5)	
Gestational week	26.22 (8.76)	29.37 (9.41)	0.125 ^a^
Pregnancy plan			
Planned	35 (87.5)	33 (82.5)	0.754 ^b^
Unplanned	5 (12.5)	7 (17.5)	
Health problems during pregnancy			
Yes	19 (47.5)	10 (25.0)	0.062 ^b^
No	21 (52.5)	30 (75.0)	
Harmful habits			
Yes	6 (15.0)	4 (10.0)	0.735 ^b^
No	34 (85.0)	36 (90.0)	
Social support during pregnancy			
Yes	34 (85.0)	32 (80.0)	0.769 ^b^
No	6 (15.0)	8 (20.0)	
Previous breastfeeding education			
Yes	9 (22.5)	13 (32.5)	0.453 ^b^
No	31 (77.5)	27 (67.5)	
Thinking that breastfeeding will be easy after birth			
Yes	35 (87.5)	34 (85.0)	0.745 ^b^
No	5 (12.5)	6 (15.0)	
Importance of breastfeeding for the baby (0–10 points)	9.85 (0.57)	9.90 (0.63)	0.713 ^a^
Importance of breastfeeding for the mother (0–10 points)	9.77 (0.89)	9.90 (0.44)	0.429 ^a^
Importance of breastfeeding for the economy (0–10 points)	9.70 (0.99)	9.70 (1.60)	1.000 ^a^

Categorical variables are presented as n (%)and continuous variables as mean (SD)

^a^ Independent-Samples t test

^b^ Chi-square test

### Prenatal breastfeeding self-efficacy scores

Baseline breastfeeding self-efficacy levels of pregnant women were found to be similar between the groups (EG 68.27 ± 13.16; CG 71.37 ± 8.22; *p* = 0.211); however, after breastfeeding education, breastfeeding self-efficacy levels in the education group were significantly higher than those in the control group (EG 83.20 ± 11.28; CG 72.72 ± 5.47; *p* = 0.000) (Table 2). According to these results, video-assisted breastfeeding education was found to be effective in increasing breastfeeding self-efficacy during pregnancy.

When the intra-group score differences were analyzed, a statistically significant improvement was found in PBSES scores after the education in the education group (*p* = 0.000), while no significant difference was found in the control group (*p* = 0.287). Breastfeeding self-efficacy score increased by an average of 14.92 points after the education given to pregnant women. This increase was significantly higher than the average increase in the control group (1.35 points) (*p* = 0.000).

**Table 2:** Comparison of the mean PBSES scores between and within groups

PBSES scores	Education group (*n* = 40)	Control group (*n* = 40)	Between groups *p*-value ^a^
Pre-test	68.27 (13.16)	71.37 (8.22)	0.211
Post-test	83.20 (11.28)	72.72 (5.47)	**0.000**
Within group p-value ^b^	**0.000**	0.287	
Difference	14.92 (15.94)	1.35 (7.91)	**0.000**

Data are presented as mean (SD)

^a^ Independent-Samples t test

^b^ Paired samples t-test

## Discussion

We conducted a randomized controlled study to determine the effect of video-supported breastfeeding education given to primiparous pregnant women on breastfeeding self-efficacy. Although there was no statistical difference in breastfeeding self-efficacy scores between the experimental and control groups at the beginning of the study, in the post-test there was a statistically significant difference in the experimental group with an increase in breastfeeding self-efficacy scores both within and between groups. This difference shows that the hypothesis “Online video-assisted breastfeeding education increases the breastfeeding self-efficacy levels of primiparous pregnant women” is confirmed. The logic of this study is to contribute to future trainings on breastfeeding in primiparous pregnant women by investigating the effect of video-supported breastfeeding training, in which the mother-baby meeting and first breastfeeding are demonstrated, on breastfeeding self-efficacy in primiparous pregnant women.

Although there are many educational studies on breastfeeding, education is usually given in the postpartum period in these studies [17, 25, 39, 40]. Many interventions to increase breastfeeding self-efficacy occur after the baby is born and begins breastfeeding. However, it is necessary to focus on increasing the breastfeeding self-efficacy of pregnant women, starting from the pregnancy period [11, 29, 41, 42]. Improving breastfeeding self-efficacy in primiparous pregnant women is even more critical as these women experience pregnancy, motherhood, and breastfeeding for the first time and are more likely to give up breastfeeding in the face of difficulties [29]. On the other hand, developing this belief before the baby is born and thus responsibilities increase may make it easier for women to start breastfeeding and breastfeed for longer periods [17]. A mother who has recently delivered is trying to recover on the one hand and to adapt to the role of motherhood on the other. In this case, it is thought that women will not be able to benefit from breastfeeding education at the desired level. Therefore, self-efficacy should be developed in the mother and prenatal breastfeeding preparation should be ensured by completing breastfeeding education during pregnancy [17].

Breastfeeding self-efficacy appears as a very important indicator of starting breastfeeding, and there are many factors that affect breastfeeding self-efficacy in primiparous pregnant women. Especially; the pregnant woman’s feeling prepared for breastfeeding, educational status, breastfeeding planning status, income level, anxiety and marital status are among the factors affecting breastfeeding self-efficacy [43]. The key word in this context is to make them feel adequate and prepared. If women believe they can breastfeed, their chances of success will increase. When looked at, self-efficacy, which is based on Bandura’s self-efficacy theory, can be intervened in supporting breastfeeding in women because it can be changed, corrected and improved [11, 17, 44]. At the same time, higher self-efficacy leads to more positive breastfeeding results [11]. In this context, it becomes important to prepare and use a training program that uses innovative approaches in improving breastfeeding self-efficacy in primiparous pregnant women. In recent years, the use of video demonstrations in breastfeeding education programs has been increasing and is more recommended [28, 32]. Our study, prepared in this direction, is, to the best of our knowledge, and aimed at improving self-efficacy the first educational study presented online to primiparous pregnant women using a video showing the first breastfeeding between mother and baby in the postpartum period.

In some hospitals in Turkey, there are prenatal classes where breastfeeding education is provided. Although breastfeeding information is provided today, as a general approach, mothers are supported to breastfeed only during their stay in the hospital after birth. This support may be about the benefits of breast milk. As far as we know, there is no routine practice in which pregnant women are educated by meeting mother and baby or demonstrating the first breastfeeding, which is the originality of this study. Online breastfeeding support and technological developments appear to increase mothers’ knowledge about breastfeeding. A meta-analysis reported that technological applications used by nurses in breastfeeding counseling increased complete breastfeeding rates and mothers’ breastfeeding self-efficacy [45, 46].

The use of online tools with current innovative approaches in education, especially the integration of breastfeeding into education by demonstrating breastfeeding, can benefit women’s breastfeeding self-efficacy in their first pregnancy [47]. Therefore, it is thought that self-efficacy scores increase by integrating video demonstration, one of the technological methods, into breastfeeding self-efficacy training programs [48]. A meta-analysis study showed that educational and supportive interventions based on innovative approaches such as e-technology such as the internet and mobile phones can significantly improve mothers’ breastfeeding attitudes [46]. In a video study conducted by showing pregnant women only once, watching the video just one time without any training did not affect the breastfeeding outcome in women. In the same study, it was recommended that video-assisted breastfeeding practices be presented within the scope of training [49]. In our study that sharing the video prepared as the most important component of the education with pregnant women may have increased the likelihood of it being watched again. A different study states that it is appropriate to use video clips in teaching lactation physiology to women [50]. Therefore, in our study, it is thought that presenting the training with video support was a driving force in the increase in breastfeeding self-efficacy. It is thought that some conditions may have an impact on this result. Although there have been positive developments in Turkey in recent years, the fact that child care and nutrition is still largely the responsibility of women, motherhood is seen as sacred [51] may have increased the motivation of primiparous pregnant women to benefit from training. On the other hand, receiving feedback from pregnant women after all sessions and monitoring their awareness may have created peer interaction by increasing the positive interaction within the group [52]. As a result, online video-supported breastfeeding education increased the perception of self-efficacy in primiparous pregnant women.

### Limitations

This study has some limitations. First of all, the results cannot be generalized to all pregnant women since the study was conducted in a single centre. Secondly, long-term effectiveness of breastfeeding education could not be evaluated in this study because there was no follow-up in the postpartum period. Thirdly, due to the Covid 19 epidemic that was ongoing in our country at the time the study was planned, the study was planned synchronously online. This situation is the realization of the education process in an online environment created additional costs due to internet access and difficulty in interaction between the educator and pregnant women.

## Conclusions

Breastfeeding self-efficacy in primiparous pregnant women was developed before birth with video-assisted breastfeeding education with online interview. It is recommended to utilize video-assisted education during pregnancy in order to increase breastfeeding success from the early postpartum period by developing breastfeeding self-efficacy during pregnancy. Future studies may investigate breastfeeding self-efficacy in primiparous pregnant women by providing face-to-face or online video support as well as hands-on training.

## Data Availability

The datasets used and/or analyzed during the current study are not publicly available due to the sensitive nature of the interviews. If someone wants to request the data from this study, the corresponding author should be contacted.

## Abbreviations

WHO: World Health Organization
UNICEF: United Nations Children’s Fund
PBSES: Prenatal Breastfeeding Self-Efficacy Scale
